# Relationship Between Odds of Reporting a Fall in the Past Year and Health Literacy Among Community-Dwelling Older Adults in Japan: A Preliminary Cross-Sectional Study

**DOI:** 10.3390/ijerph23070875

**Published:** 2026-07-05

**Authors:** Akihiko Murayama, Ken Hirukawa, Tomoyuki Shinohara

**Affiliations:** 1 Department of Physical Therapy, Faculty of Rehabilitation, Gunma University of Health and Welfare, Maebashi Plaza Genki 21 6-7F, 2-12-1 Hon-machi, Maebashi 371-0023, Gunma, Japan; 2 Department of Rehabilitation, Geriatrics Research Institute and Hospital, 3-26-8 Oodomo-machi, Maebashi 371-0847, Gunma, Japan; 2132041@takasaki-u.ac.jp; 3 Department of Physical Therapy, Faculty of Health Care, Takasaki University of Health and Welfare, 27 Nakaorui-machi, Takasaki 370-0033, Gunma, Japan; shinohara-t@takasaki-u.ac.jp

**Keywords:** falls, health literacy, community-dwelling older adults

## Abstract

**Highlights:**

**Public health relevance—How does this work relate to a public health issue?**

- As population aging continues, the prevalence of fall-related injuries, including fractures and head injuries, increases.
- Among community-dwelling older adults, health literacy is associated with falls.

**Public health significance—Why is this work of significance to public health?**

- Lack of consensus regarding the definition and conceptual framework of health literacy limits measurement and comparison across studies.
- Focusing on community-dwelling older adults in Japan, a super-aging society, is valuable for developing future international models.

**Public health implications—What are the key implications or messages for practitioners, policy makers and/or researchers in public health?**

- Considering the physical, cognitive, and health literacy status of community-dwelling older adults is important.
- Various approaches should be established to assess the risk of falls among community-dwelling older adults.

**Abstract:**

Background/Objectives: Research on falls among community-dwelling older adults has revealed limited knowledge regarding health literacy. This study aimed to evaluate health literacy in community-dwelling older adults in Japan and to clarify its relationship with the odds of reporting a fall in the past year. Methods: A preliminary cross-sectional study was conducted from January 2024 to May 2025, involving 218 community-dwelling older adults. The study collected data on age, sex, presence of multiple diseases, use of the long-term care insurance system, the Brief-Balance Evaluation Systems Test (Brief-BESTest), the Rapid Dementia Screening Test (RDST), the Communicative and Critical Health Literacy (CCHL) scale, and fall reports in the past year. Results: After excluding four participants, 214 participants (age, 76.9 ± 6.4 years; 61 men and 153 women) were included in the analysis. Of these, 43 (20.1%) participants had reported a fall. A binary logistic regression analysis was performed, adjusting for confounding factors (age, RDST score, and Brief-BESTest score). The reporting of a fall in the past year (falls/non-falls) was used as the dependent variable, and the CCHL score as the independent variable. The results showed that the CCHL score was associated with the reporting of a fall (odds ratio, 0.61; 95% confidence interval, 0.40–0.93). Conclusions: These findings suggest that higher CCHL scores, reflecting better communicative and critical health literacy, are associated with lower odds of reporting a fall. However, because of the cross-sectional design of this study, no causal relationship can be inferred. Future longitudinal studies with larger sample sizes are needed to clarify the temporal relationship between health literacy and falls, examine whether this association is causal, and investigate whether the relationship is linear or whether clinically meaningful threshold effects exist.

## 1. Introduction

As the global population ages, primary healthcare systems face increasing challenges in addressing the complex health and social needs of older adults [1]. With continued population aging, the incidence of fall-related injuries, including fractures and head injuries, increases [2,3]. Falls also substantially reduce quality of life and impose a considerable economic burden on healthcare systems and society in high-income countries [4]. Public health priorities have shifted from reducing mortality to preventing disability, and falls are a major contributor to disability burden among older adults, resulting in substantial years lived with disability and reduced healthy life expectancy [5,6].

In recent years, there has been an increase in research on identifying risk factors for falls among older adults [7]. A previous study classified these risk factors into intrinsic (individual) and extrinsic (environmental) factors [8]. Identifying individuals with high fall risk and implementing preventive strategies to avoid severe injuries, such as fractures, could reduce the escalating costs associated with medical and nursing care [9].

Therefore, establishing effective strategies to reduce fall risk among community-dwelling older adults is paramount. However, clearly defined prevention strategies for this population remain limited. Community-dwelling older adults encounter various environmental hazards that differ from those in hospital or institutional settings [10,11]. A previous study on community-dwelling older adults highlighted the need for multifaceted assessments and interventions for fall prevention [12]. However, the limited resource availability for fall prevention interventions and the need for optimized resource allocation must be considered in parallel [13].

Various factors may be associated with fall risk, including health literacy; however, evidence remains limited. Health literacy refers to the ability to obtain, understand, and use health-related information. Its importance in public health has attracted international attention in recent years [14]. Previous studies have demonstrated that health literacy strongly predicts health behaviors, health-related outcomes, and general well-being [15,16,17,18]. Community-dwelling older adults with high health literacy have been shown to engage in regular exercise more frequently than those with low health literacy [19]. Higher health literacy has also been associated with a lower risk of frailty progression [20]. Moreover, health literacy related to movement and behavioral practices influences walking ability, activities of daily living, and fall risk among community-dwelling older adults [21,22]. Despite increasing attention to health literacy in fall prevention strategies, limited evidence exists regarding its relationship with falls and impact on fall risk [23].

Therefore, we hypothesized that health literacy would be related to fall occurrence among community-dwelling older adults in Japan. Two reasons supported the selection of this target population. In Japan, the term “older adults” refers to individuals aged ≥65 years. As of September 2025, the geriatric population in Japan was 36.19 million, accounting for 29.4% of the total population. Japan has the highest proportion of older adults among 200 countries and regions with populations of ≥100,000 [24]. According to the 2022 Basic Survey on National Livelihoods conducted by the Ministry of Health, Labour and Welfare, fractures and falls accounted for 13.0% of older adults requiring nursing care, ranking third [25]. Three years prior, fractures and falls ranked fourth, indicating a growing public health problem [26].

The Programme for the International Assessment of Adult Competencies revealed that Japanese individuals of all ages have higher numerical reasoning and reading comprehension skills compared with those of other nationalities [27]. However, inadequate health literacy is highly prevalent in Japan. Comparisons of health literacy in Japan with that in European and Asian countries showed that a high percentage of respondents found items related to information evaluation and decision-making difficult. The limited availability of reliable, publicly accessible websites and the absence of standardized information sources contribute to low health literacy in Japan [28,29].

Focusing on community-dwelling older adults in Japan, a leading super-aging society, is highly valuable for developing future international health models. This preliminary study offers novel insights by examining fall risk from the perspective of health literacy. Specifically, this study aimed to evaluate health literacy in community-dwelling older adults in Japan and to clarify its relationship with the odds of reporting a fall in the past year. Given the scarcity of knowledge in this field, we believe the study findings will provide a foundation for future large-scale longitudinal studies and inform new fall prevention interventions.

## 2. Materials and Methods

This preliminary cross-sectional study included older adults aged ≥65 years living in Takasaki City, Gunma Prefecture, Japan. Takasaki City is the most populous municipality in Gunma Prefecture. According to the most recent basic resident register, the total population of the city is 368,196, of which 105,696 residents are classified as older adults. This study did not constitute a complete population survey. This initiative was implemented in collaboration with community-based organizations, such as community centers and salons. Data were collected from a public community center—a community-based space established by the local government where activities such as volunteer-led exercise classes for older adults are held.

Between January 2024 and May 2025, 237 individuals took part in this study; of these, 19 individuals under the age of 65 were excluded, resulting in 218 community-dwelling older adults. All participants provided written informed consent. The study was conducted in accordance with the principles of the Declaration of Helsinki and was approved by the Research Ethics Committee of the Takasaki University of Health and Welfare (approval numbers 2358 (date of approval 9 January 2024) and 2457 (date of approval 9 January 2025)). The present study was conducted using data collected under the same ethics-approved research protocol investigating falls, balance function, frailty, and health-related factors in community-dwelling older adults. Health literacy was one of the variables specified in the approved protocol and participant consent.

The inclusion criteria were as follows: (1) independence in activities of daily living; (2) ability to independently visit various locations, including local salons; and (3) ability to walk indoors without assistive devices, such as canes. Study variables included age, sex, presence of multiple diseases (defined as the presence of two or more chronic diseases [30]), use of the long-term care insurance system, reporting a fall in the past year, and Brief-Balance Evaluation Systems Test (Brief-BESTest), Rapid Dementia Screening Test (RDST), and Communicative and Critical Health Literacy (CCHL) scores. Fall categories were classified as no falls, one fall, and two or more falls in the past year. Classification by the number of falls is also possible; however, the boundary between single and repeated falls differs depending on the research objective. Considering that previous epidemiological studies have commonly analyzed outcomes based on the presence or absence of falls [31], this study adopted a binary classification. Data on the study variables (age, sex, presence of multiple diseases, use of the long-term care insurance system, and reporting a fall in the past year) were collected using a questionnaire developed by the authors to assess the participants’ general characteristics.

Considering its application in local practice, the standard version of Brief-BESTest [32] was applied, which allows for the evaluation of all balance systems within a short time. The Brief-BESTest includes eight items derived from six sections of the BESTest. The reliability and validity of this tool for balance assessment have been demonstrated [33]. Each item is scored on a 4-point scale (0–3), with a total score ranging from 0 to 24 points. One item is selected from each section of the BESTest [34]: I, biomechanical limitations; II, stability limits/verticality; III, anticipatory postural control; IV, postural response; V, sensory orientation; and VI, gait stability. The assessment requires approximately 10 min to complete. Lower Brief-BESTest scores have been associated with increased fall risk [35].

Cognitive decline is a risk factor for falls [36]. Therefore, the RDST [37] was administered as a brief assessment of cognitive function. The RDST consists of two components: the first is a verbal fluency task using the category “supermarket,” in which participants list as many purchasable items as possible within 1 min; the second is a number conversion task in which participants convert Arabic numerals into written words and vice versa. The test requires approximately 3–5 min to administer and score. An RDST score of ≤9 suggests possible mild cognitive impairment (MCI), whereas a score of ≤7 indicates more pronounced cognitive decline.

The CCHL scale [38] was developed for the general population, independent of specific diseases, based on functional, communicative, and critical health literacy constructs proposed by Ishikawa et al. [39] for individuals with diabetes. Responses are recorded on a 5-point Likert scale: 1 (strongly disagree), 2 (somewhat disagree), 3 (neither agree nor disagree), 4 (somewhat agree), and 5 (strongly agree). The mean score of the five items is calculated, with higher scores indicating higher health literacy. CCHL evaluates higher-order dimensions of health literacy beyond functional health literacy. According to Nutbeam [40], health literacy includes three levels: functional, interactive, and critical. Functional health literacy refers to the basic ability to read and write effectively in daily life; it is assessed through a self-assessment questionnaire that assumes the absence of reading or writing difficulties. Interactive health literacy refers to the ability to obtain information through communication with others and to act independently based on acquired knowledge and advice. Critical health literacy refers to the ability to critically analyze information and apply it to manage daily situations. The present study used the CCHL scale because interactive and critical health literacy are emphasized in public health and health promotion more frequently than functional health literacy [41,42,43].

The sample size was determined based on previous systematic reviews and meta-analyses reporting an approximate falls prevalence of 25% among community-dwelling older adults [44,45]. Based on the premise that approximately one in four individuals experience a fall, the required sample size was calculated using an allocation ratio of 1:4 (fall group/non-fall group) between groups. Given that pwr in R does not provide a direct argument for unequal group ratios, the uniroot function was combined with pwr to solve the equation under the condition that n2 was four times n1. Owing to the limited availability of relevant prior research and the difficulty in estimating the effect size, a moderate effect size (Cohen’s *d* = 0.5) was assumed based on Cohen’s criteria [46]. Using a significance level of 5% and 80% power when applying a two-tailed test, the required sample size was determined to be 40 participants in the fall group and 160 in the non-fall group, resulting in a total sample of 200 participants.

Descriptive statistics were used to describe participant characteristics. Binary logistic regression analysis was performed with fall reporting in the past year (falls/non-falls) as the objective variable, following the rule that the number of events should be at least 10 times the number of explanatory variables when divided into binary outcomes [47]. Overparameterized logistic regression models with too many explanatory variables relative to the number of events can produce unrealistically large odds ratios and excessively wide confidence intervals—a phenomenon called overfitting [48]. Explanatory variables were not selected based on statistically significant differences observed in univariate comparisons between the fall and non-fall groups. A hypothesis-driven approach guided variable selection because an association between health literacy and falls among community-dwelling older adults was hypothesized. Variable selection based solely on univariate analyses reflects characteristics of the current dataset and may not be generalizable to other populations [49]. Therefore, covariates were selected a priori based on previous evidence and clinical relevance rather than statistical significance in the present sample. Specifically, age, balance function, and cognitive function were included because they have been identified as important risk factors for falls in the World Falls Guidelines [50]. All statistical analyses were performed using EZR (EZR on R Commander ver. 1.70; Jichi Medical University Saitama Medical Center, Saitama, Japan), which is a graphical user interface for R (The R Foundation for Statistical Computing, Vienna, Austria). More precisely, it is a modified version of R commander designed to add statistical functions frequently used in biostatistics [51], with a significance level of 5%.

## 3. Results

**Figure 1:**
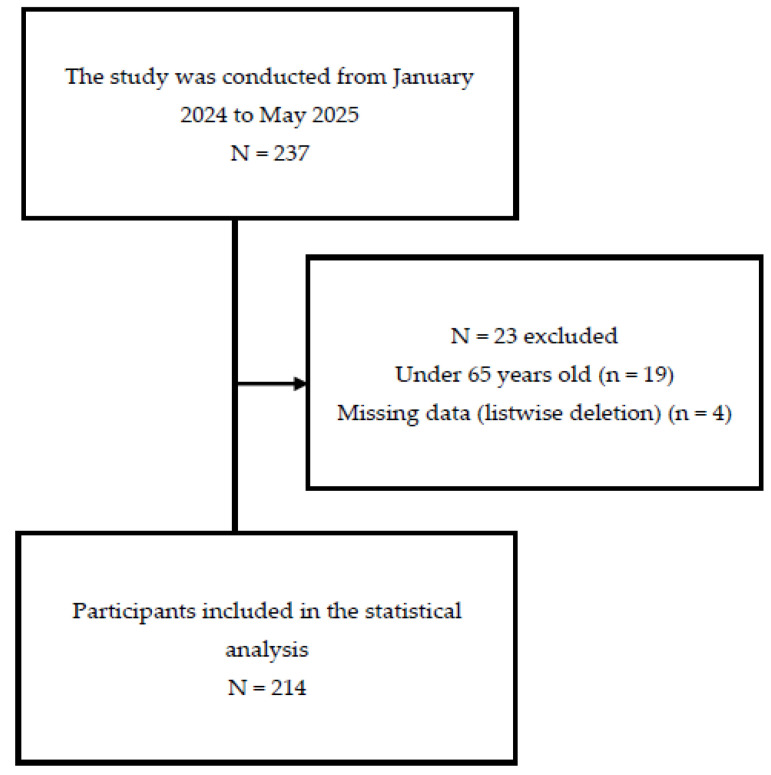
Flowchart of the participant selection process.

Excluding four participants with missing survey responses (listwise deletion), 214 participants (mean age, 76.9 ± 6.4 years; 61 men and 153 women) were included in the analysis (Figure 1).

The participants were classified into fall (n = 43; 20.1%) and non-fall (n = 171; 79.9%) groups based on the reporting of a fall in the past year. Of the 43 participants in the fall group, 29 reported one fall and 14 reported two or more falls.

The distribution of the study variables in the fall and non-fall groups, respectively, was as follows: age, 79.8 ± 6.2 and 76.2 ± 6.2 years; sex, 31 and 122 women; multiple diseases, 14 and 51 participants; use of long-term care insurance, 2 and 12 participants; RDST score, 9.8 ± 2.6 and 10.2 ± 2.4; Brief-BESTest score, 16.0 ± 4.5 and 18.7 ± 4.2; and CCHL score, 3.2 ± 0.7 and 3.6 ± 0.8 (Table 1).

Binary logistic regression analysis was performed using the forced entry method on the fall group (n = 43). The reporting of a fall in the past year (falls/non-falls) was used as the dependent variable, and the CCHL score was used as the independent variable. Taking this into consideration, three adjustment variables were prespecified: age, balance function (Brief-BESTest score), and cognitive function (RDST score). Considering the limited size of the fall group and the potential risk of model overfitting, additional established risk factors, including sex, multiple diseases, and long-term care insurance status, were not included in the final model. Binary logistic regression showed that the CCHL score was associated with the dependent variable (odds ratio, 0.61; 95% confidence interval, 0.40–0.93). Furthermore, the Brief-BESTest score, which was included for adjustment, was independently associated with the dependent variable (odds ratio, 0.88; 95% confidence interval: 0.79–0.97). The result of the χ^2^ test for the model was significant (*p* < 0.001). The results of the Hosmer–Lemeshow test indicated good model fit (*p* = 0.894) (Table 2).

## 4. Discussion

This study suggests that higher CCHL scores, reflecting better communicative and critical health literacy, are associated with lower odds of reporting a fall. Previous studies [52,53] have recognized the role of health literacy in fall prevention among community-dwelling older adults. Suwannakul et al. [54] found that higher health literacy is associated with important fall prevention behaviors, including regular exercise, safer home hygiene practices such as the use of Western-style toilets, and wearing appropriate footwear. However, the lack of consensus regarding the definition and conceptual framework of health literacy limits measurement and comparison across studies. Therefore, mechanisms underlying the relationship between health literacy and falls remain poorly understood.

In this study, higher CCHL scores, reflecting better communicative and critical health literacy, are associated with lower odds of reporting a fall. Although balance function (Brief-BESTest) was also associated with falls, this relationship has been consistently demonstrated in previous studies and was therefore regarded as an adjustment factor rather than a primary finding. Importantly, the association between communicative and critical health literacy and falls remained after adjustment for age, cognitive function (RDST), and balance function (Brief-BESTest), suggesting that the observed association cannot be fully explained by these established fall-related factors.

The present study exclusively included older adults with the ability to walk indoors unassisted and navigate outdoors independently. Therefore, older adults with mild walking difficulties and a high risk of falls may have been excluded. Accordingly, the results should not be interpreted as applicable to all community-dwelling older adults, but rather as limited to highly independent individuals.

Recent years have seen increased emphasis on comprehensive health promotion addressing the overall lifestyle of community-dwelling older adults, and educational interventions have demonstrated effectiveness [55]. Exercise interventions for fall prevention have been associated with a small-to-moderate reduction in concerns about falls immediately after the intervention among community-dwelling older adults. However, these effects diminish after several months [56]. Considering these findings, a sustainable training program combining expert-supervised outdoor exercise with telecoaching may be effective in improving balance and muscle strength in older adults. This approach may represent a safe, effective, and efficient intervention strategy for increasing physical activity levels and maintaining high adherence to training in older adults [57].

The CCHL is a self-administered questionnaire that can be easily completed. Adding this instrument to existing assessments for community-dwelling older adults is unlikely to substantially increase assessment time or participant burden. Future studies should identify which components of the CCHL most effectively predict falls. The results of the current study may provide alternative options for older adults who have difficulty maintaining exercise programs or are reluctant to engage in exercise initially. However, this interpretation remains speculative, and no clear reports have been published to date. Therefore, further research will be necessary.

This study has some limitations. First, participant selection was not randomized, and sex and age matching based on regional distribution was not possible; therefore, the representativeness of the sample is unclear. However, the number of variables included in the final model was appropriate. Second, although sex, multiple diseases, and long-term care insurance status are recognized risk factors for falls, these variables were not included because of the limited number of fall events and the need to avoid model overfitting. Consequently, residual confounding cannot be ruled out. Furthermore, key determinants influencing health literacy and fall incidence, such as education level, socioeconomic status, living situation, physical activity level, visual impairment, medication use/polypharmacy, and psychological state (including depression), were not considered; thus, residual confounding remains an important concern. Third, the reporting of a fall in the past year was evaluated based on the participants’ self-reports, and therefore, the influence of recall bias cannot be completely ruled out. Previously, recall bias (vague memory or underreporting) when confirming fall history has been identified in community-dwelling older adults [58]. Nonetheless, the participants of the current study were generally able to live independently in the community: the mean RDST score was 10.1 ± 2.4 points, exceeding the cutoff value for suspected MCI [37]. Therefore, the participants’ recall of a fall in the past year was likely sufficiently preserved. However, because the interviews were not conducted on a weekly or monthly basis, there are limitations to understanding the reporting of a fall in the past year. Finally, because this study was preliminary and employed a cross-sectional design, it can only demonstrate associations and cannot establish temporal or causal relationships.

Despite these limitations, we believe this study makes a notable contribution to the current knowledge in this field by supporting the relationship between health literacy and the odds of reporting a fall in the past year among community-dwelling older adults in Japan. Future longitudinal studies with larger sample sizes are needed to clarify the temporal relationship between health literacy and falls, examine whether this association is causal, and investigate whether the relationship is linear or whether clinically meaningful threshold effects exist.

## 5. Conclusions

This study found a significant association between health literacy and the odds of reporting a recent fall among community-dwelling older adults in Japan. These findings, obtained from community-dwelling older adults in Japan, one of the world’s most rapidly aging societies, may contribute to the development of future international health models for fall prevention. However, given the preliminary cross-sectional nature of this study, future large-scale longitudinal studies are needed to clarify this association. This preliminary study also provides a basis for future investigations into whether a clinically meaningful threshold of health literacy exists for fall prevention and whether the association between health literacy and falls is linear across the full range of health literacy.

## Figures and Tables

**Table 1:** Basic characteristics of the participants.

	Overall	Fall Group	Non-Fall Group
	(n = 214)	(n = 43)	(n = 171)
Age, Mean ± SD	76.9 ± 6.4	79.8 ± 6.2	76.2 ± 6.2
Sex, (men/women), n (%)	61 (28.5)/153 (71.5)	12 (27.9)/31 (72.1)	49 (28.7)/122 (71.3)
Multiple diseases, (presence/absence), n (%)	65 (30.4)/149 (69.6)	14 (32.6)/29 (67.4)	51 (29.8)/120 (70.2)
Long-term care insurance, (use/not use), n (%)	14 (6.5)/200 (93.5)	2 (4.7)/41 (95.3)	12 (7.0)/159 (93.0)
RDST (score), Mean ± SD	10.1 ± 2.4	9.8 ± 2.6	10.2 ± 2.4
Brief-BESTest (score), Mean ± SD	18.2 ± 4.4	16.0 ± 4.5	18.7 ± 4.2
CCHL (score), Mean ± SD	3.5 ± 0.8	3.2 ± 0.7	3.6 ± 0.8

RDST, Rapid Dementia Screening Test; Brief-BESTest, Brief-Balance Evaluation Systems Test; CCHL, Communicative and Critical Health Literacy; SD, standard deviation.

**Table 2:** Results of the binary logistic regression analysis.

Independent Variable	Odds Ratio	95% Confidence Interval	*p*-Value
CCHL (score)	0.61	0.40–0.93	0.023
Age	1.05	0.98–1.13	0.154
RDST (score)	1.12	0.95–1.33	0.172
Brief-BESTest (score)	0.88	0.79–0.97	0.014

CCHL, Communicative and Critical Health Literacy; RDST, Rapid Dementia Screening Test; Brief-BESTest, Brief-Balance Evaluation Systems Test. Binary logistic regression analysis was performed using the forced entry method with the following conditions: reporting of a fall in the past year (falls/non-falls) was the dependent variable; the CCHL score was the independent variable; age, RDST, and Brief-BESTest scores were adjustment variables. Hosmer–Lemeshow test indicated good model fit (*p* = 0.894).

## Data Availability

The datasets generated and analyzed during the current study are not publicly available due to ethical restrictions and participant consent limitations. The study participants did not provide consent for public sharing of individual-level data. Information regarding the study variables, data dictionary, and statistical analysis procedures is available from the corresponding author [A.M.] upon reasonable request and subject to approval by the institutional ethics committee.

## Abbreviations

The following abbreviations are used in this manuscript:

Brief-BESTest	Brief-Balance Evaluation Systems Test
RDST	Rapid Dementia Screening Test
CCHL	Communicative and Critical Health Literacy
